# Intracranial management of HER-2 overexpression breast cancer with extensive volume or symptomatic brain metastases

**DOI:** 10.3389/fonc.2024.1386909

**Published:** 2024-07-01

**Authors:** Juan Li, Junjie Zhen, Ruyu Ai, Mingyao Lai, Hui Wang, Linbo Cai

**Affiliations:** Department of Oncology, Guangdong Sanjiu Brain Hospital, Guangzhou, China

**Keywords:** breast cancer, brain metastasis, HER-2 positive, radiotherapy, targeted drug

## Abstract

**Objectives:**

This study aimed to evaluate the impact of high intracranial burden and symptomatic presentation of brain metastases on treatment outcomes in patients with HER-2 positive breast cancer. Through a retrospective analysis, we explored the intracranial responses following the application of HER-2 targeted therapy alone or in combination with other modalities and further elucidated the relationship between treatment efficacy, intracranial progression-free survival (PFS), overall survival (OS), and the burden of intracranial lesions and symptomatic presentations.

**Methods:**

A retrospective analysis was conducted on cases of HER-2 overexpressing breast cancer patients with brain metastases. Clinical records were reviewed to extract patient demographics, treatment modalities, and intracranial disease characteristics. Intracranial tumor burden was quantified at diagnosis and post-initial treatment. High intracranial tumor burden was defined as either total metastatic volume >15 cc, or the largest lesion >3 cm. Responses were assessed using established criteria. The correlation between intracranial disease parameters and intracranial progression-free survival (PFS) and overall survival (OS) was determined.

**Results:**

The study comprised 65 patients with HER-2 overexpression breast cancer and brain metastases. Symptomatic presentation was observed in 69.2% of patients at the diagnosis of brain metastases. Treatment with HER-2 target therapy alone or in combination with other modalities resulted in substantial intracranial responses, with 81.5% achieving at least a partial response at 3 months from therapy initiation. Median intracranial PFS and OS for patients with high intracranial burden were 9 and 22 months, respectively. Patients with high intracranial burden and symptomatic presentation at diagnosis demonstrated worse PFS and OS to those with lower burden and absence of symptoms (p < 0.05 for each).

**Conclusions:**

Her-2 overexpressing breast cancer and brain metastases face significant challenges, particularly those with high intracranial tumor burden, which correlates with poorer outcomes and higher incidence of leptomeningeal metastasis. Most patients responded positively to initial therapies, especially anti-HER-2 treatments combined with radiotherapy. Larger tumors necessitated more comprehensive treatment approaches, such as WBRT and SRS. Key factors influencing intracranial tumor control included the Ki-67 index, intracranial tumor burden, and continuous use of HER-2 targeted therapy post-diagnosis.

## Introduction

Patients with breast cancers overexpressing human epidermal growth factor receptor 2 (Her-2) bear an increased risk for developing brain metastases, attributed to longer survival times (1, 2) and inherent genomic modifications (3). Brain metastases and systemic disease are often managed distinctly due to the poor Central Nervous System (CNS) penetration by systemic agents. Recommended local therapies for brain metastases include surgical resection, stereotactic radiosurgery (SRS), or whole-brain radiation therapy (WBRT), depending on the imaging characteristics of the brain metastases and the severity of neurological symptoms.

Herceptin (trastuzumab), a monoclonal antibody therapy, has been increasingly used alone or in combination with local therapy for managing brain metastases in Her-2 overexpressing breast cancer patients (4). This treatment has demonstrated improved CNS penetrance and has shown its clinical effectiveness against brain metastases (3, 5, 6). Current guidelines suggest that treatment with a systemic agent showing good CNS penetrance can be considered in selected patients with small and asymptomatic brain metastases (7). Yet, the management of breast cancer brain metastases largely differs based on institutional experience and clinician preferences (8). In the case of large or numerous brain metastases, the preferred treatment modality isn’t established, and such situations historically suggest worse intracranial control and survival (9, 10).

Despite extended survival times, large retrospective studies have observed no significant difference in overall survival between SRS and WBRT (11). Another study reported improved outcomes for patients who received SRS along with trastuzumab in the upfront management of brain metastases from Her-2 overexpressing breast cancer, but the selection criteria for SRS eligibility remain unclear, subjecting their results to selection bias (12). Moreover, no study to our knowledge has specifically addressed outcomes for those at higher risk due to larger or symptomatic brain metastases.

## Materials and methods

In this study, we aim to correlate clinical and imaging factors associated with survival in patients with brain metastases from Her-2 overexpressing breast cancer and treated with trastuzumab. We retrospectively identify and review cases of Her-2 overexpression breast cancer with brain metastases. Clinical characteristics and treatment data are abstracted from the medical records, and brain metastases are contoured to calculate total volume of disease at diagnosis and after initial therapy. High intracranial load is defined according to specific criteria, and intracranial response is gauged according to RANO criteria (13) at the patient level. Our purpose is to determine prognostic factors to help guide future studies in defining the optimal upfront therapy for this cohort of patients.

### Data collection

With approval from the institutional review board, we reviewed the medical records of patients with Her-2 overexpressing breast cancer, diagnosed with brain metastases between March 2013 and April 2022, and treated at our institution. Inclusion criteria for this study include individuals aged 18–65 years diagnosed with breast cancer, specifically HER-2 positive, and confirmed to be free of brain metastases through cranial magnetic resonance imaging. Exclusion criteria encompass individuals with incomplete clinical data, male patients with breast cancer or bilateral breast cancer, those with poor overall health status unable to tolerate treatment, and individuals with concomitant other tumors or cranial diseases.

Collected data included the year of birth, gender, Karnofsky performance status (KPS), score of Zubrod-ECOG-WHO performance status (ZPS), presence of neurological symptoms, dates of breast cancer and brain metastases diagnosis, and any Her-2 targeted therapy use. Brain lesions were contoured in a Velocity treatment planning system (Varian Medical Systems, Palo Alto, CA) to calculate maximal tumor diameter and total volume of disease (cc). High intracranial burden was defined as either > 10 brain metastases, a volume of brain metastases > 15 cc, or the largest lesion > 3 cm. These cutoffs were selected based on eligibility criteria from the Trans-Tasman Radiation Oncology Group OUTRUN trial (NCT03497767). T1, post-contrast magnetic resonance imaging (MRI) revealing the first instance of brain metastases for each patient was used for contouring by a senior resident in radiation oncology. Follow-up MRI after initial treatment (>2 months and<4 months post-treatment) was similarly contoured. Small lesions no longer identifiable were marked as a 100% volumetric response on the post-treatment scan. Treatment characteristics, such as surgical resection, systemic agents including trastuzumab, SRS, and WBRT used in the upfront and salvage settings were recorded. Toxicities were scored according to the Common Terminology Criteria for Adverse Events (CTCAE) Version 5.0 (14).

### Treatment

Decisions regarding brain metastases treatment were made by treating physicians, often in collaboration with multidisciplinary breast and brain metastases tumor boards. Systemic therapy was administered by medical oncology based on standards of care at the time of brain metastasis diagnosis. Treatment options for brain metastasis include Whole Brain RT (WBRT) for cases with more than 10 metastases or certain histologies, while Stereotactic Radiosurgery (SRS) is preferred for cases with 10 or fewer metastases.For patients treated with SRS, this was performed on the Novalis treatment System (Brainlab, Heimstetten, Germany). Treatment plans were generated from thin slice MRI that were merged with a stereotactic CT scan. The prescription dose was based on tumor size, location, and prior radiation therapy. The final plan was developed in collaboration with a neurosurgeon, radiation oncologist, and medical physicist. The dose selection for stereotactic radiosurgery (SRS) in the treatment of metastatic tumors is based on the findings of the Radiation Therapy Oncology Group (RTOG) 90–05 trial, which determined the maximum tolerated doses as follows: 24 Gy for tumors measuring less than 2.0 cm, 18 Gy for tumors measuring 2.1–3.0 cm, and 15 Gy for tumors measuring 3.1–4.0 cm. For patients who received WBRT, memantine and hippocampal avoidance were used at physician discretion. Follow-up MRI was performed at intervals based on physician discretion. Our institutional standard for MRI surveillance is typically every 3 months for those treated with trastuzumab and SRS and without symptoms.

### Outcome analysis

The latest available post-treatment MRI within four months of treatment was used to compute volumetric response. Response was determined at the patient level, not by individual lesion. Using the available volumetric data, we utilized the RANO suggested partial volumetric response definition as a 65% or more decrease in the sum volume of target lesions, with stable or decreased corticosteroid use, and stable or improved clinical status (15). A complete response was the disappearance of all lesions, no corticosteroid use, and stable or improved clinical status. Progressive disease was defined as an increase greater than 20% in the diameter of treated lesion(s), or new brain lesions. Freedom from intracranial progression was defined as time from brain metastases diagnosis to intracranial progression (per RANO criteria) as determined by CNS imaging, and did not use death as an event. Intracranial progression-free survival (IC-PFS) was defined as time from brain metastasis diagnoses until intracranial progression (per RANO criteria) or death. Overall survival was defined as the time from brain metastases diagnosis until death from any cause. These were summarized using Kaplan–Meier methodology. Index cancer diagnosis date was determined by the original pathology report. Cox regression univariable and multivariable analyses were performed to identify prognostic clinical or treatment characteristics. SPSS Statistics (International Business Machines Corporation, Armonk, NY) software and the R statistical computing language (R Foundation for Statistical Computing) was used for analyses. Results from univariable analyses with a trend toward significance (p<0.10) were included in the multivariable models.

## Results

### Patient and treatment characteristics

A total of 65 eligible patients with Her-2 overexpressing breast cancer were identified. Median follow-up from initial brain metastases diagnosis was 24 months (range 1.9–55 months). Brain metastases and breast cancer diagnosis were synchronous in 14 (21.5%) cases, whereas the remaining were metachronous at a median of 51 months (range 1.6–156.8 months) after breast cancer diagnosis. In 51 patients with measurable lesion size, the patients with high intracranial tumor burden (n = 21) had a median of 3 brain metastases, 26.5 cc volume, and 2.9 cm maximal tumor diameter. Patients with low intracranial burden (n=30) had a median of 3 brain metastases, 4.6 cc volume, and 1.2 cm maximal tumor diameter. Neurological symptoms were present in 54.4% of cases at brain metastasis diagnosis.

Six patients underwent resection of dominant brain metastasis, three with high burden and three with low burden. Fifty-two cases (80%) received anti-HER-2 targeted therapy, including 44 cases treated with monoclonal antibodies such as trastuzumab or pertuzumab, and 1 case treated with single-agent tyrosine kinase inhibitors such as lapatinib or pyrotinib. Additionally, 7 patients received treatment with a combination of two types of drugs.

Patients with larger diameter tumors were more likely to receive whole brain radiotherapy and SRS in addition to Her-2 targeted therapy (odds ratio = 2.3per cm maximal tumor size, p = 0.03 on regression analysis). All patients received radiotherapy during the treatment process. Among them, 60% of brain metastases received treatment, including radiotherapy, as part of their initial treatment. Following progression of the brain disease after initial treatment, radiotherapy was administered to all patients. Regarding the radiotherapy approach for patients, 38 cases (58.5%) received whole-brain radiation therapy (WBRT) combined with stereotactic radiosurgery (SRS), 23 cases (35.4%) received SRS alone, and 4 cases (6.2%) received WBRT alone (**Table 1**).

**Table 1:** Patient characteristics.

	n or median	% or range
Patients	65	
Clinical characteristics		
Age at brain metastases diagnosis (years)	Median 50	(27-71)
ECOG at brain metastases diagnosis		
0-1	41	63.1%
2-4	24	36.9%
Brain metastases at diagnosis		
No	59	90.8%
Yes	6	9.2%
Neurologic Symptoms		
No	20	30.8%
Yes	45	69.2%
Largest metastasis tumor size (cm)	2.8	0.8-5.5
Total brain metastases volume (cc)	15	0.35-90.8
Number of metastases	2	1-26
Initial Treatment characteristics		
System treatment + radiotherapy	28	43.1%
System treatment + surgery	7	10.8%
System treatment without local treatment	14	21.5%
Radiotherapy alone	11	16.9%
Surgery alone	5	7.7%
Radiotherapy approach		
SRS+WBRT	38	58.5%
SRS	23	35.4%
WBRT	4	6.2%
Upfront Surgery		
No	53	81.5%
Yes	12	18.5%
Her2 targeted agents	52	80%
Monoclonal antibody (Trastuzumab or Pertulumab)	44	67.8%
TKI(Lapatinib/Pyrotinib)	1	1.5%
Both	7	10.8%

### Treatment response at 3 months

**Figure 1:**
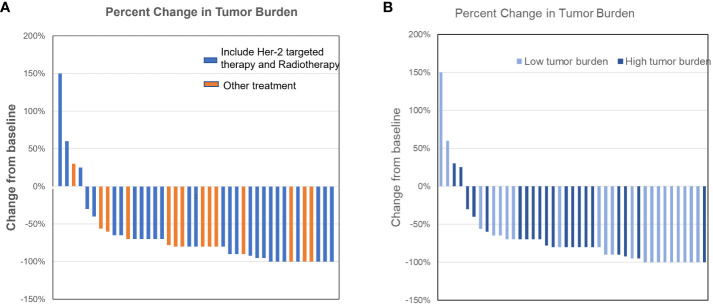
**(A)** The tumor regression status under different treatment methods. **(B)** The tumor regression status under different tumor burdens after initial treatment.

An intracranial response (partial or complete response) to any initial therapy was observed in 57 (87.7%) of 65 patients with post-treatment brain images available at approximately 3 months. Response per RANO criteria was complete in 16 (24.6%), partial in 41 (63.1%), stable in 2 (3.1%), and progressive disease in 6 (9.2%) of the 65 patients. Salvage/consolidative SRS (n = 2) and WBRT (n = 1) were provided to those patients with early progressive disease (**Figure 1**).

Full volumetric response for patients receiving trastuzumab as part of their therapy is shown in **Figure 1**. Specifically, two cases with Her-2 overexpressing breast cancer, treated with trastuzumab alone had progressive disease at the first post-treatment scan. 2 cases of progression were following surgery and the other 2 were with trastuzumab + WBRT.

### Occurrences of leptomeningeal metastasis

Among 65 patients, 3 patients were suspected of leptomeningeal metastasis at initial diagnosis, while 19 patients were diagnosed with leptomeningeal metastasis during the course of the disease. Among these, 15 cases (78.9%) occurred in the high tumor burden group, and 4 cases (21.1%) occurred in the low tumor burden group.

### Factors influencing intracranial tumor control

**Figure 2:**
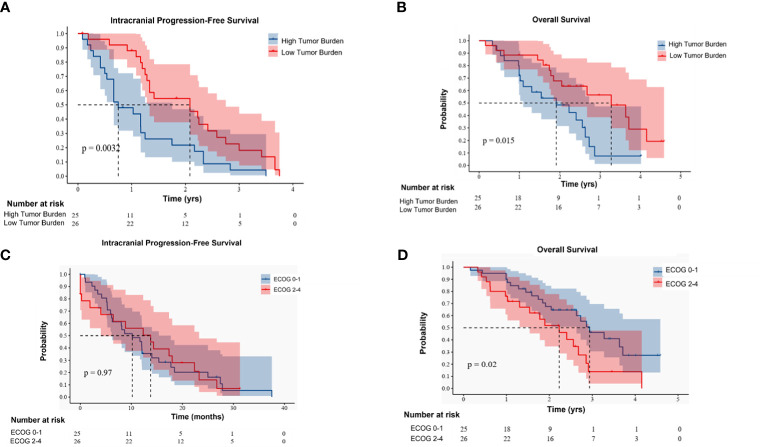
**(A, B)** respectively represent the effects of different intracranial tumor burdens on intracranial progression-free survival (PFS) and overall survival (OS). **(C, D)** respectively represent the effects of Eastern Cooperative Oncology Group (ECOG) performance status on intracranial PFS and OS.

Using a multivariable Cox regression model, it was analyzed that the primary tumor Ki-67 index, intracranial tumor burden, and continuous use of HER-2 targeted therapy after the occurrence of brain metastasis are factors influencing intracranial tumor control (**Table 2**). Both intracranial tumor burden and ECOG performance status significantly affect intracranial progression-free survival and overall survival. Higher tumor burdens and poorer performance status are associated with worse outcomes in terms of both PFS and OS (**Figure 2**).

**Table 2:** Analysis of factors associated with intracranial progression free survival following diagnosis of brain metastases.

		IC-PFS	UVA	MVA			
		Median(m)	P value	P value	HR	95%CI for HR	
						Lower	Upper
Age	<40yrs	11.0					
	>40yrs	14.1	0.493	0.507	0.89	0.123	2.084
ER	Positive	17.1					
	Negative	12.0	0.448	0.833	2.559	0.39	2.13
PR	Positive	17.1					
	negative	13.6	0.389	0.084	2.656	0.169	1.118
Ki-67	≥50%	7.0					
	<50%	17.1	0.065	0.012	2.989	0.116	0.761
Her2 targeted therapy	Yes	20					
	No	14	0.037	0.046	3.998	1.028	15.324
ECOG score	0-1	20					
	2-4	13.6	0.97	0.204	1.617	0.292	1.3
Tumor burden	Low	20					
	High	9.0	0.003	0.025	4.991	1.129	6.411

Tumor burden means the intracranial tumor.

## Discussion

Patients with HER-2 positive breast cancer treated with first-line Trastuzumab, have shown improved survival rates. However, a longer survival period also raises the likelihood of intracranial metastases (15). Our study explored the correlation between prognosis and intracranial metastatic burden, as well as neurological symptoms. We observed a correlation between the intracranial metastatic burden, the presence of neurological symptoms, and overall survival. Early use of radiotherapy in combination with anti-HER-2 targeted drugs for detected intracranial metastases can significantly reduce tumor volume, thereby enhancing the patients’ quality of life. Hence, regardless of intracranial metastatic burden and neurological symptoms, the use of radiotherapy along with anti-HER-2 targeted drugs may be considered in the upfront setting.

Previous breast cancer research has indicated a correlation between the total volume of brain metastases and survival rates (1, 16). In our study, we aimed to establish a connection between the tumor response rate of breast cancer brain metastases and the control of intracranial tumors. Despite this, across the overall population, initial treatment of metastatic tumors yielded satisfactory outcomes. No significant differences were observed in tumor regression within three months across all treatment groups. This result may be due to sample selection bias, as a majority of our selected patients underwent local treatment (surgery or cranial radiotherapy) after the onset of brain metastases, and most did not discontinue systemic treatment. This validates the effectiveness of continuous systemic treatment combined with more aggressive local treatment after the occurrence of brain metastases in HER-2 positive breast cancer (17, 18). This differs from the effective control of gene mutation-driven lung cancer brain metastases simply with TKI treatment. We found that patients with a larger intracranial tumor burden had shorter intracranial control times and were more likely to develop meningeal metastases in subsequent follow-ups. Currently, the treatment of meningeal metastases remains a challenge in the therapy of breast cancer brain metastases (19).

During the course of targeted therapy, patients with HER-2 positive breast cancer tend to be more susceptible to brain metastases. Following the occurrence of brain metastases in these patients, the progression of the tumor is generally rapid, leading to a larger tumor burden. This subsequently results in a higher incidence of neurological symptoms. In the clinical trials of drugs targeting HER-2 positive breast cancer, patients presenting with accompanying neurological symptoms are often excluded (20). This presents a significant gap in our understanding and management of this patient group, as their treatment experience and outcomes may differ from those without neurological symptoms. In our data set, there were 12 patients who were treated with TKI drugs such as pyrotinib and all belonged to the high tumor burden group. This provides invaluable real-world experience and evidence of the use of these novel drugs in patients presenting with neurological symptoms. These observations emphasize the need for disease management strategies that consider the unique clinical characteristics and treatment challenges associated with brain metastases in HER-2 positive breast cancer. Additionally, it underscores the potential role of novel TKI therapies in managing this patient population, particularly those with a high tumor burden and neurological symptoms. Further studies on the efficacy of such treatments and strategies for managing neurological symptoms are warranted.

The criteria for a high tumor burden include either a large tumor volume or a high number of tumors. Both scenarios necessitate different treatment strategies. Stereotactic Radiosurgery (SRS) and Whole Brain Radiation Therapy (WBRT) are two commonly employed treatment modalities for brain metastases (21). For larger volume brain metastases, the treatment of choice typically leans towards local therapies such as stereotactic radiosurgery (SRS) or surgical intervention. SRS is typically completed in a shorter duration, often requiring only a single treatment session or a few fractions, making it convenient for patients. In cases of brain metastases located in functional areas, neurological symptoms are often present. Unlike surgery, which may carry the risk of permanent functional damage, SRS can rapidly alleviate symptoms in these patients. In contrast, for patients with more numerous tumors, whole-brain radiotherapy is usually employed. Whole-brain radiotherapy is also the basic principle when meningeal metastases occur.

Trastuzumab, an anti-HER2 monoclonal antibody, represents a class of large molecule monoclonal antibodies. Previous studies have shown that trastuzumab has difficulty penetrating the blood-brain barrier, resulting in lower cerebrospinal fluid drug concentrations within the central nervous system. This could explain why the effectiveness of trastuzumab is reduced when used as a standalone treatment for brain metastases. On the other hand, drugs like pyrotinib, a type of TKI, demonstrate a greater ability to penetrate the blood-brain barrier. Whether using pyrotinib alone can delay the initiation of cranial radiotherapy, especially whole-brain radiotherapy, and thereby preserve patients’ cognitive abilities, warrants further clinical trials. These findings highlight the complexities and nuances of treating HER-2 positive breast cancer patients with brain metastases, and underscore the need for more tailored and effective therapeutic strategies.

In our study, most HER2-positive patients opted for Trastuzumab or Pertuzumab as their initial treatment. Upon the occurrence of brain metastases, oncologists face the decision of whether to continue using these drugs or to combine them with TKI or chemotherapy. As it is generally believed that brain metastases are difficult to control with Trastuzumab alone, and extracranial targeted drugs can continue to be effective, it is usually recommended to use local therapy to control brain metastatic lesions. Anti-HER2 drugs can be continued or replaced with small molecule TKIs.

Radiotherapy is believed to open up the blood-brain barrier in the meninges, and it’s worth noting whether the concentration of drugs such as Trastuzumab could effectively increase after radiotherapy. This potential increase could enhance the efficacy of these drugs in treating brain metastases. However, this hypothesis requires further investigation and if proven, could influence treatment strategies for patients with HER2-positive breast cancer and brain metastases.

The therapeutic approach following initial treatment failure in brain metastases poses a significant challenge, as there is currently no consensus on second and third-line treatments. Apart from traditional methods such as re-radiation and repeat surgery, the efficacy of intrathecal injection of targeted drugs remains to be further explored. Intrathecal administration of drugs, which delivers therapy directly into the spinal canal, could potentially increase drug concentrations at the site of the brain metastases and thereby enhance treatment effectiveness. However, the safety, feasibility, and overall impact on patient outcomes of this approach require further investigation. This highlights the pressing need for more research and clinical trials in this area to establish a standardized approach for the management of brain metastases following the failure of initial treatment. Ultimately, the goal is to improve the prognosis and quality of life for patients with brain metastases.

### Limitation

Several limitations in our study need to be considered (1): This is a single-center retrospective study, which may lead to selection bias (2). The patients included in our study spanned a decade. In recent years, there have been significant changes in the systemic treatment of brain metastases in HER2-positive breast cancer. The ultimate medical choices of patients depend on the treatment plan of different medical teams, the patients’ disease and economic factors, and the availability and tolerance of drugs, all of which may cause potential bias in treatment (3). Systemic treatment for HER2-positive brain metastases patients in this study was not handled in our department, making long-term follow-up and comprehensive assessment of intracranial lesions challenging, such as the evaluation of neurotoxicity and cognitive levels.

## Conclusion

Her-2 overexpressing breast cancer patients with brain metastases face significant challenges in disease management. The study identified a high intracranial tumor burden in a substantial subset of patients, which was associated with poorer outcomes, including a higher incidence of leptomeningeal metastasis. Despite these challenges, the majority of patients exhibited a positive response to initial therapies, particularly those involving anti-HER-2 targeted treatments combined with radiotherapy. Patients with larger tumors were more likely to receive comprehensive treatment approaches, such as whole-brain radiotherapy (WBRT) combined with stereotactic radiosurgery (SRS). Key factors influencing intracranial tumor control included the Ki-67 index of the primary tumor, the extent of intracranial tumor burden, and the continuous use of HER-2 targeted therapy after the diagnosis of brain metastasis. These findings underscore the importance of personalized and aggressive treatment strategies to improve intracranial tumor control and patient outcomes.

## Data availability statement

Users may be required to cite the dataset or provide acknowledgment to the data provider when using the dataset. Requests to access the datasets should be directed to lijuangz@foxmail.com.

## Ethics statement

This study was approved by the Ethics Committee of Guangdong Sanjiu Brain Hospital. Written informed consent was obtained from all patients included in the study.

## Author contributions

JL: Writing – original draft, Writing – review & editing. JZ: Conceptualization, Data curation, Writing – original draft. RA: Formal analysis, Funding acquisition, Writing – original draft. ML: Investigation, Methodology, Project administration, Writing – original draft. HW: Resources, Software, Writing – original draft. LC: Validation, Visualization, Writing – original draft.
